# Reflex Epilepsy from Dental Caries

**Published:** 1891-02-07

**Authors:** 


### REFLEX EPILEPSY FROM DENTAL CARIES.

Dr. Badowski reports an interesting case of epilepsy in a girl aged sixteen, the attacks recurring several times a day, and persisting for nine months in spite of treatment with bromides, arsenic, quinine, &c., until his attention being called to the carious condition of the first upper molar tooth on the right side, and the first lower molar on the left, which, though not causing her any pain, appeared to be the seat of a peculiar sensation *(aura)* before the attacks, he advised their extraction. This was done, and when he wrote six months later she had not had a single attack.

Cases like this tend to confirm an opinion for which we have long contended, heterodox though it may appear, that epilepsy has little or no right to a place in nosology as a special disease, but is merely an expression for a certain combination or aggregate of reflex phenomena, induced by a multiplicity of causes. This is generally admitted of infantile convulsions, the exciting causes of which are mostly such as would induce headaches in the adult, and which occasionally pass into confirmed epilepsy. If so, pathologists will still search in vain for an essential causative lesion in epilepsy, finding only any or no conditions in their *post-mortem* examinations of the nervous centres. Had this girl been sixteen months instead of years of age, her case would have been properly described as one of teething convulsions, though the vast majority of those so-called are really due to improper feeding, and correspond to the sick-headaches of gastric disturbance, or of constipation in older patients.

